# A case of metastatic carcinoma of anal fistula caused by implantation from rectal cancer

**DOI:** 10.1186/s40792-015-0125-2

**Published:** 2015-12-11

**Authors:** Rina Takahashi, Ryosuke Ichikawa, Singo Ito, Kosuke Mizukoshi, Shun Ishiyama, Kiichi Sgimoto, Yutaka Kojima, Michitoshi Goto, Yuichi Tomiki, Takashi Yao, Kazuhiro Sakamoto

**Affiliations:** Department of Coloproctological Surgery, Faculty of Medicine, Juntendo University, 2-1-1 Hongo, Bunkyo-ku, Tokyo, 113-8421 Japan; Department of Human Pathology, Faculty of Medicine, Juntendo University, 2-1-1 Hongo, Bunkyo-ku, Tokyo, 113-8421 Japan

**Keywords:** Metastatic carcinoma of anal fistula, Colorectal cancer, Implantation

## Abstract

This case involved an 80-year-old man who was seen for melena. Further testing revealed a tubular adenocarcinoma 50 mm in size in the rectum. In addition, an anal fistula was noted behind the anus along with induration. A biopsy of tissue from the external (secondary) opening of the fistula also revealed adenocarcinoma. Nodules suspected of being metastases were noted in both lung fields. The patient was diagnosed with rectal cancer, a cancer arising from an anal fistula, and a metastatic pulmonary tumor, and neoadjuvant chemotherapy was begun. A laparoscopic abdominoperineal resection was performed 34 days after 6 cycles of mFOLFOX-6 therapy. Based on pathology, the rectal cancer was diagnosed as moderately differentiated adenocarcinoma, and this adenocarcinoma had lymph node metastasis (yp T3N2aM1b). There was no communication between the rectal lesion and the anal fistula, and a moderately differentiated tubular adenocarcinoma resembling the rectal lesion was noted in the anal fistula. Immunohistochemical staining indicated that both the rectal lesion and anal fistula were cytokeratin 7 (CK7) (−) and cytokeratin 20 (CK20) (+), and the patient’s condition was diagnosed as implantation of rectal cancer in an anal fistula.

In instances where an anal fistula develops in colon cancer, cancer implantation in that fistula must also be taken into account, and further testing should be performed prior to surgery.

## Background

A handful of studies have reported primary cancer arising from a chronic anal fistula [1], but implantation of tumor cells in an anal fistula is rare. Only 27 cases of this condition have been reported since the report by Guiss et al. [2]. As reported here, the current authors encountered a case in which cancer cells migrating from rectal cancer were implanted in an anal fistula.

## Case presentation

This case involved an 80-year-old man. At age 76, a stent had been placed in the man’s left coronary artery to treat coronary arteriosclerosis. As of August 2013, the man was seen by his previous physician for melena. Colonoscopy revealed rectal cancer, and the patient was referred to our hospital for further testing and treatment.

**Fig. 1:**
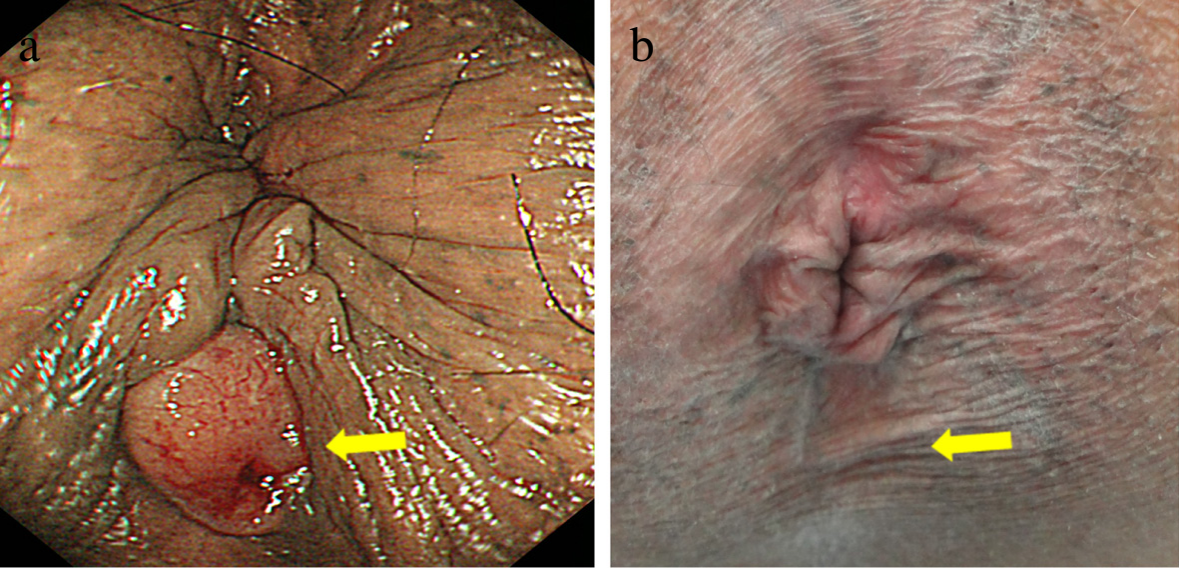
**a** The second external opening of an anal fistula was noted behind the anus. **b** After NAC, the second external opening of an anal fistula behind the anus was no longer evident

Upon examination, a mass was not palpated during a rectal examination, but the external opening of an anal fistula was noted behind the anus (Fig. 1a). The fistula was straight and could be palpated induration. There was granulation tissue at the external opening, but the patient had no subjective symptoms like pain or discharge of pus, and the timing when the anal fistula developed was unclear.

Blood chemistry results revealed anemia with Hb of 10.0 g/dl. The level of the tumor marker CEA was elevated (26.9 ng/ml).

**Fig. 2:**
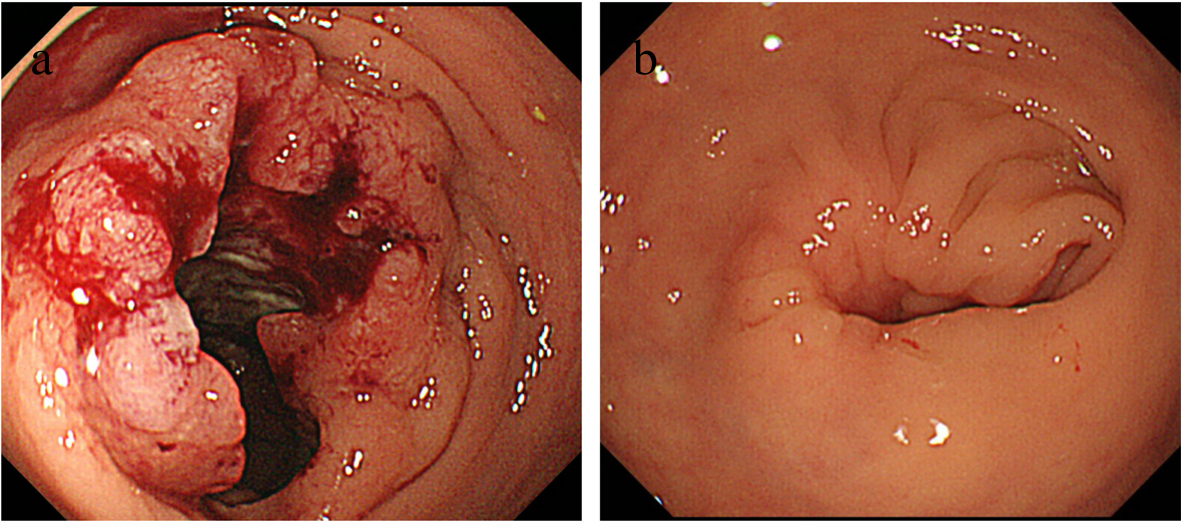
**a** Colonoscopy revealed the lower margins of a tumor in the rectum 7 cm from the anal verge. **b** Colonoscopy after NAC. Ulcerated folds were less prominent

**Fig. 3:**
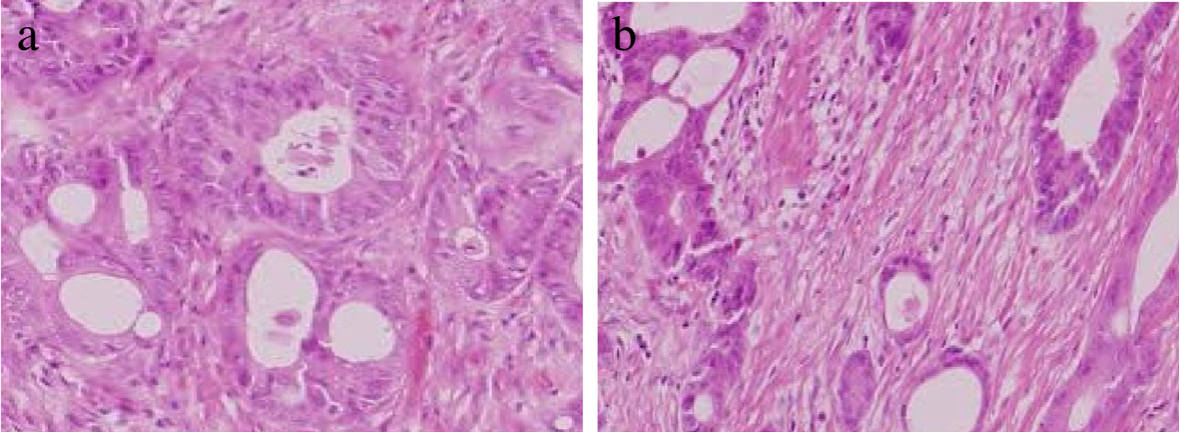
**a** Histopathology indicated that the rectal lesion was a moderately differentiated adenocarcinoma (H&E ×40). **b** Histopathology indicated that the anal fistula was found to be a moderately differentiated tubular adenocarcinoma (H&E ×40)

Colonoscopy revealed the lower margins of a tumor in the rectum 7 cm from the anal verge. This lesion was 50 mm in size and covered two-thirds of the circumference of the rectum (Fig. 2a). Based on a biopsy, the tumor was diagnosed as well-differentiated tubular adenocarcinoma (Fig. 3a). In addition, the fistula was also diagnosed as adenocarcinoma based on a biopsy (Fig. 3b).

A CT scan revealed thickening of the rectal wall with contrast enhancement and swelling of the mesorectum. The scan also revealed five nodules of 12 mm in size in both lungs that were suspected of being metastases.

Thus, the patient’s condition was diagnosed as rectal cancer, a cancer arising from an anal fistula, and a metastatic pulmonary tumor, and neoadjuvant chemotherapy (NAC) was initiated with 6 cycles of mFOLFOX-6 therapy. The patient received oxaliplatin 75 mg/m^2^, 5-fluorouracil (320 mg/m^2^ by bolus infusion and 2000 mg/m^2^ by continuous infusion), and leucovorin 200 mg/m^2^.

After NAC, the level of the tumor marker CEA dropped to 4.6 ng/ml. Colonoscopy revealed a tumor covering one-fourth of the circumference of the rectum 7 cm from the anal verge. Ulcerated folds were less prominent (Fig. 2b). The tumor was diagnosed as well-differentiated tubular adenocarcinoma based on a biopsy. The second external opening of an anal fistula behind the anus was no longer evident (Fig. 1b).

After NAC, a CT scan revealed reduced thickening of the rectal wall and shrinkage of mesorectum lymph nodes. Nodules in both lung fields shrank, and tumor shrinkage according to the RECIST criteria was 34 %.

Adverse events due to chemotherapy were not noted, and a laparoscopic abdominoperineal resection was performed 34 days after the final cycle of chemotherapy. During perineal resection, adequate margins were obtained around the anal fistula and the rectum was dissected while palpating the fistula. Part of the levator ani near the fistula was laparoscopically resected, but efforts were made to preserve as much of the levator ani as possible on the opposite side of the anal fistula. The end stoma was created through the extraperitoneal route. The anal fistula was a simple fistula.

Histopathology indicated that the rectal lesion was a moderately differentiated adenocarcinoma, and the adenocarcinoma had lymph node metastasis (yp T3N2aM1b, yp stage IV B). The pathological response about effective of NAC was grade 1b (*Japanese Classification of the Colorectal Carcinoma*, Seventh Edition) [3].

**Fig. 4:**
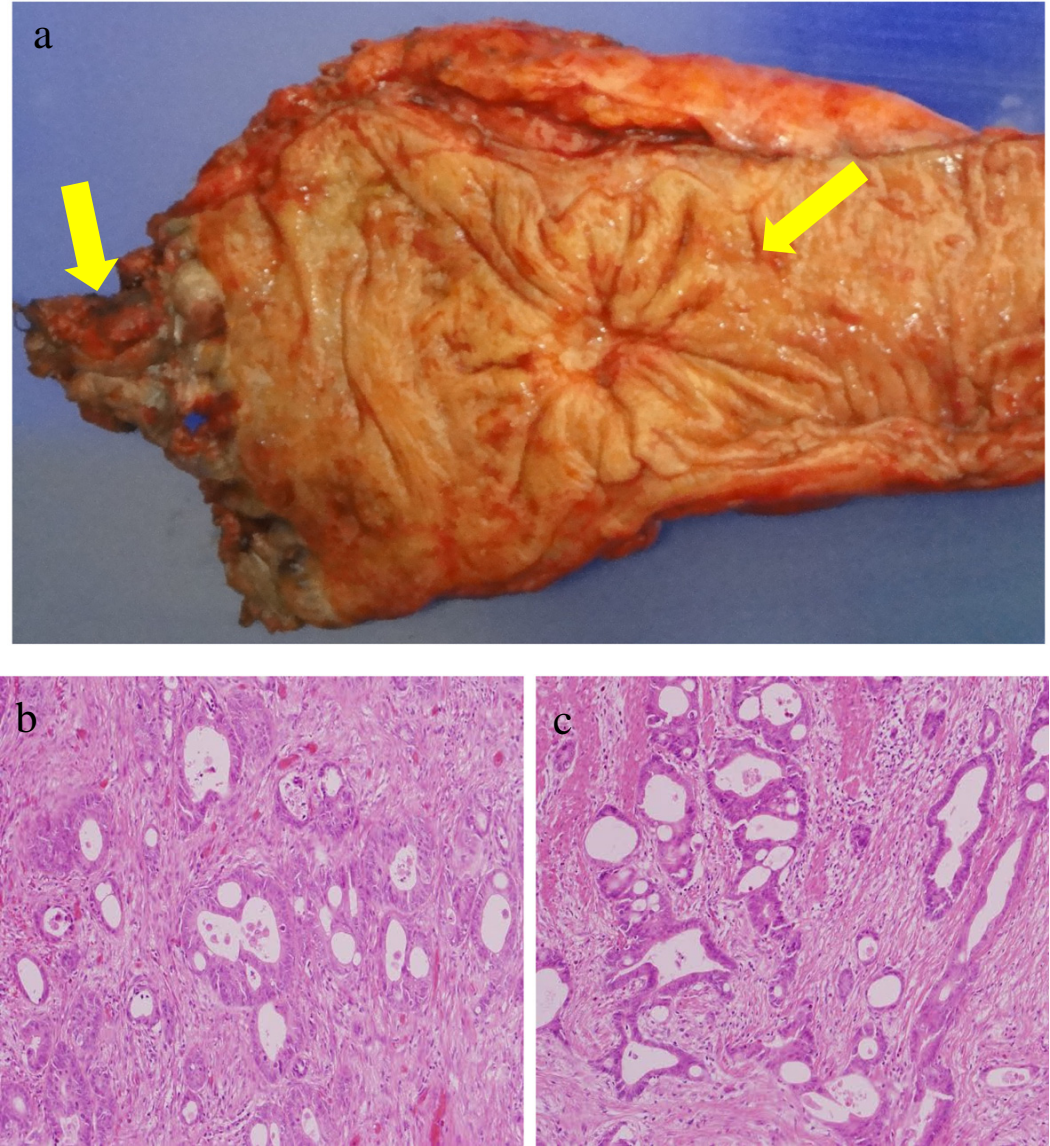
**a** There is no communication with rectal lesion and the anal fistula **b** Histopathology indicated that the rectal lesion was a moderately differentiated adenocarcinoma (H&E ×20). **c** The anal fistula was found to be a moderately differentiated tubular adenocarcinoma resembling the rectal lesion (H&E ×20)

**Fig. 5:**
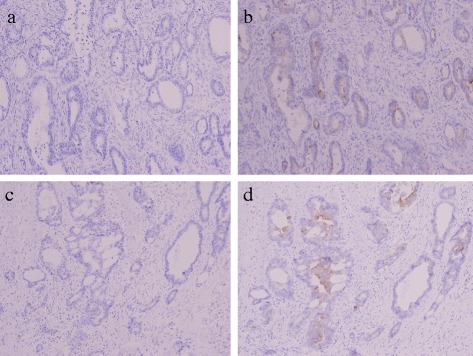
Immunohistochemical staining (×20). Rectum lesion stained CK7 (−) **(a)** CK20 (+) **(b)** and the fistula stained CK7 (−) **(c)** and CK20 (+) **(d)**

The length of anal fistula was 15 mm; the internal opening was found at the behind anal crypts. The anal fistula was found to be a moderately differentiated tubular adenocarcinoma resembling the rectal lesion, but communication with the rectal lesion was not noted (Fig. 4). In immunohistochemical staining, both the rectal lesion and the anal fistula stained negative (−) with antibodies to cytokeratin 7 (CK7) and positive (+) with antibodies to cytokeratin 20 (CK20) (Fig. 5).

The patient’s postoperative course was satisfactory, and the patient was discharged on day 14 of hospitalization. As of 14 months postoperatively, the patient continues to receive chemotherapy on an outpatient basis. New metastases have not been noted, and there has been no change in the lung metastases and patient’s status.

### Discussion

A persistent anal fistula with recurrent inflammation is known to lead to primary cancer, and such cancer develops in 0.1 % of all anal fistulae [4]. As Skir [5] and Mclntyre et al. [6] noted, the diagnostic criteria for primary cancer arising from an anal fistula are as follows: (1) recurring inflammation of the fistula for at least 10 years, (2) increasing pain and induration at the fistula, (3) mucus secretion, (4) no primary cancer at the rectum and anus, and (5) the fistula has an opening in the anal canal or an anal crypt.

That said, cancer implantation in an anal fistula occurs when cancer cells migrating from advanced colon cancer are implanted in an anal fistula. The basis for this diagnosis is when implanted cells have the same histologic type as the primary tumor. In addition, immunohistology can provide clues to differentiate cancer implantation in an anal fistula from other types of cancer. A combination of CK7 and CK20 can facilitate this determination. Loy et al. [7] reported that the vast majority of colon cancers (80 %) are CK7 (−)/CK20 (+) while some (16 %) are CK7 (+)/CK20 (+) and a few (4 %) are CK7 (−)/CK20 (−). In six of seven cases of primary cancer arising from an anal fistula, those cancers were CK7 (+)/CK20 (−) [8]. In instances where colon cancer and cancer arising from an anal fistula are both present, the primary cancer must be differentiated from metastases. In cases previously encountered by the current authors, this cancer had a histologic type resembling colon cancer, and immunohistochemical staining indicated that both the lesion and the fistula stained negative for CK7 and positive for CK20. Thus, a diagnosis of implantation of colon cancer in an anal fistula was made.

According to Umpleby et al. [9], colon cancer cells shed into the intestinal lumen are implanted in injured mucosa. An inflammatory response to a stimulus (like a bacterial infection) or wound healing activates cancer cell growth, presumably resulting in cancer implantation in an anal fistula [10, 11].

Cancer implantation in an anal fistula was first reported by Guiss et al. [2], though similar implantation of colorectal cancer cells in a hemorrhoidectomy wound [12] and implantation of rectal cancer in the anal canal due to anal injury occurring during insertion of a circular stapler [13] have also been reported. Thus, the potential for cancer implantation must also be taken into account in patients with colon cancer who develop an anal fistula and in patients who have undergone surgery for an anal disorder. Such patients must be carefully followed prior to surgery. When the anus is handled during surgery, avoiding and preventing injury to the anoderm is crucial. When cancer arising from an anal fistula is diagnosed, the proximal intestine should be examined with metastasis of colon cancer in mind.

There are 27 reported cases of implantation of colon cancer in an anal fistula, which include cases previously encountered by the current authors [14–16]. In most of those cases, the primary tumor was found in the sigmoid colon or the rectum (2 in the descending colon, 13 in the sigmoid colon, 10 in the upper rectum, and 2 in the lower rectum). In about half of the 27 cases, cancer arising from an anal fistula was diagnosed prior to resection of the primary tumor, and an abdominoperineal resection was performed. A handful of reports have described performing only local resection when cancer arising from an anal fistula was diagnosed after resection of the primary tumor [17, 18]. Regardless, there are no reports of local recurrence after surgery. In the event of cancer implantation in an anal fistula, the cancer must be resected with adequate margins.

In this case, a large tumor was noted along with implantation of colorectal cancer in an anal fistula, so preoperative chemotherapy was performed. The tumor shrank as a result, and the tumor was completely removed. The fistula was also removed, albeit to a limited extent. In addition, implantation of cancer cells and pulmonary metastases were noted, so systemic treatment was provided in the form of preoperative chemotherapy. The tumor responded well to chemotherapy, and chemotherapy was continued postoperatively. As a result, SD has been maintained. Thus, the treatment chosen was appropriate.

## Conclusions

The current authors encountered a case in which cancer arising from an anal fistula was diagnosed based on a biopsy prior to surgery. In that case, cancer implantation in that fistula was similarly diagnosed based on immunostaining after surgery. In instances where an anal fistula develops in colon cancer, cancer implantation in that fistula must also be taken into account, and further testing should be performed prior to surgery.

## Consent

Written informed consent was obtained from the patient for publication of this case report and any accompanying images. A copy of the written consent is available for review by the Editor-in-Chief of this journal.
